# Machine learning insights into the antioxidant and biomolecular shielding effects of polyphenol-rich 18 date palm pit extracts

**DOI:** 10.1016/j.fochx.2025.102480

**Published:** 2025-04-19

**Authors:** Nashi K. Alqahtani, Tareq M. Alnemr, Hoda A.S. Farag, Rania Ismail, Hosam M. Habib

**Affiliations:** a Department of Food and Nutrition Sciences, College of Agricultural and Food Sciences, King Faisal University, P.O. Box 400, Al-Ahsa 31982, Saudi Arabia; b Date Palm Research Center of Excellence, King Faisal University, P.O. Box 400, Al-Ahsa 31982, Saudi Arabia; c Faculty of Computer Science & Engineering, Alamein International University (AIU), Alamein City 5060310, Egypt; d Research & Innovation Hub, Alamein International University (AIU), Alamein City 5060310, Egypt

**Keywords:** Date pits, Phytochemicals, Machine learning, XGBoost model, Antioxidants, DNA damage protection, Protein damage protection

## Abstract

This study investigated the antioxidant and biomolecular shielding effects of polyphenol-rich extracts from 18 date palm pit varieties. Phytochemical profiling revealed significant varietal differences, with catechin (358.48 mg/100 g in Maghool) and gallic acid (34.55 mg/100 g in Khalas) as dominant compounds. Antioxidant assays demonstrated robust activity, with Shikat alkahlas showing 64.10 % DPPH inhibition and Maghool exhibiting the highest FRAP value (190.57 mmol Fe^2+^/100 g). Enzyme inhibition varied widely: Maghool inhibited tyrosinase by 65.40 %, while Shabebe showed 42.21 % α-amylase inhibition. Protective effects against DNA and BSA damage were pronounced in Sagay (39.99 % supercoiled DNA retention) and Jabri (98.95 % BSA protection). An XGBoost model predicted antioxidant, enzyme inhibitory, and protective activities with up to 92.57 % accuracy, identifying catechin and cinnamic acid as key predictors. These findings underscore the potential of date pits as sustainable sources of bioactive compounds for nutraceutical and pharmaceutical applications.

## Highlights

• Comprehensive analysis of 18 date palm pit varieties.
• XGBoost model predicts bioactivities with high accuracy.
• Catechin and cinnamic acid were identified as key predictors.
• Sustainable source for functional food, nutraceutical, and pharmaceutical use.

## 1 Introduction

Date palm (*Phoenix dactylifera* L.), a cornerstone of agriculture in arid and semi-arid regions, holds immense global significance, with annual production exceeding 9.5 million metric tons (Habib, El-Fakharany, Souka, et al., 2022; Kamal et al., 2022). Leading producers such as Egypt (1.7 million tons), Saudi Arabia (1.6 million tons), and Iran (1.0 million tons) underscore its economic and nutritional value (FAO STAT, 2022). While dates are renowned for their rich profile of vitamins, minerals, and dietary fiber, their seeds (pits), constituting 6–15 % of fruit weight, remain underutilized and are often discarded as agricultural waste (Habib & Ibrahim, 2009). However, recent studies highlight date pits as a potent source of polyphenols, including flavan-3-ols and proanthocyanidins, with concentrations surpassing those in grapes and tea (Habib, Platat, AlMaqbali, & Ibrahim, 2014). These bioactive compounds exhibit antioxidant, anti-inflammatory, enzyme-inhibitory properties, anti-diarrheic, anti-diabetic, anti-lipidemic, hypoglycemic, tonic, and aphrodisiac agents, offering the potential to mitigate oxidative stress-linked disorders such as diabetes, neurodegeneration, and cardiovascular diseases (Ayatollahi, 2019; Hilary et al., 2021).

The development of natural antioxidants has gained significant attention as a promising alternative to synthetic antioxidants. In this context, it is important to consider green chemistry principles, and recent advances in sustainable extraction methods, such as ultrasound-assisted deep eutectic solvents (Marinaccio et al., 2024, Marinaccio et al., 2025), highlight eco-friendly approaches for polyphenol recovery. Research shows that polypeptides have strong antioxidant activity.

Despite their promise, the bioactivity of date pit extracts varies significantly across cultivars due to genetic, environmental, and postharvest factors (Alam et al., 2023; Hosseiny et al., 2023; Ouamnina et al., 2024; Toubali & Meddich, 2024). For instance, polyphenol-rich varieties demonstrate superior radical scavenging (e.g., DPPH, ABTS) and metal-reducing (FRAP) capacities, while others excel in inhibiting enzymes like α-amylase (glucose regulation), acetylcholinesterase (cognitive health), and tyrosinase (melanin synthesis) (Habib, El-Fakharany, Souka, et al., 2022; Hilary et al., 2021; Platat & Habib, 2014).

Recent advancements in food science have positioned date pits as sustainable ingredients for functional food innovation. Studies demonstrate their successful incorporation into baked goods (e.g., muffins, cookies, and gluten-free bread) to enhance fiber content and bioactive retention while improving textural properties (Elkatry et al., 2024). For instance, date pit powder has been used as a partial flour substitute in bread, increasing polyphenol levels by up to 30 % without compromising sensory acceptability (Platat et al., 2015). Similarly, date seed oil, rich in oleic and lauric acids, has been explored as a stable lipid source in cosmetics and fortified snacks (Nwachukwu et al., 2025). In beverages, roasted date pits serve as a caffeine-free coffee alternative, delivering antioxidant capacities comparable to traditional brews (Al-Khalili et al., 2023; Kiesler et al., 2024; Nwachukwu et al., 2025). Nevertheless, in processed cheese, date pit powder has been used as a partial substitute for fat of up to 20 % to enhance the texture and increase the bioactive (Alqahtani et al., 2023).

Machine learning (ML) emerges as a transformative tool to bridge this gap. By leveraging computational models like XGBoost, ML can decode complex structure-activity relationships, predict bioactivities from phytochemical profiles, and identify key biomarkers (Joshi et al., 2024; Kim et al., 2024). While ML has been applied to antioxidant discovery in other plants, its integration with date pit research is unprecedented (Jung et al., 2024; Li et al., 2024).

This study employed a multi-faceted approach to: characterize the phytochemical profiles of 18 date pit varieties, evaluate their antioxidant, enzyme-inhibitory, and biomolecule-protective activities, correlate bioactive compounds with observed bioactivities, and develop an ML model to predict bioactivity from phytochemical composition.

## 2 Materials and methods

### 2.1 Plant material and preparation

Pits from the 18-date variety of date palm (*Phoenix dactylifera* L.), (Khalas, Barhe, Lulu, Shikat alkahlas, Sokkery, Bomaan, Sagay, Shishi, Maghool, Sultana, Fard, Maktoomi, Naptit Saif, Jabri, Kodary, Dabbas, Raziz and Shabebe), were utilized in this study. Five kilograms of fully ripe dates were purchased from the local market. The pits were initially soaked in water to facilitate the removal of any residual date flesh, followed by thorough washing. After cleaning, the pits were air-dried and ground into a coarse powder using a crushing hammer mill (Joyal, Machinery Co., Ltd., Shanghai, China). Further refinement was achieved using a ZYM Ultrafine Powder Mill (Joyal Machinery Co., Ltd., Shanghai, China). The powder was then passed through a 0.5-mm screen using a Udy cyclone mill (UDY Corporation, Rome Ct, Fort Collins, CO 80524, United States). The resulting fine powder was sieved into two fractions using screens with openings of 0.5 mm and 0.25 mm. The fraction with a particle size of 0.25 mm was selected for extraction.

### 2.2 Study materials

All analytical grade chemicals used in this study were from Millipore Sigma Chemical Co. (St. Louis, MO, USA), except ferrous sulfate heptahydrate (FeSO₄·7H₂O), hydrochloric acid (HCl), and pBR322 plasmid DNA, which were obtained from Biolabs (Ipswich, MA, USA). These chemicals were: 1,1-diphenyl-2-picrylhydrazyl (DPPH^•^), 4-aminobenzenesulfonic acid, 4-hydroxy-3-methoxy benzoic acid, 5,5′-dithiobis-(2-nitrobenzoic acid) (DTNB), 2,2′-azobis(2-amidinopropane) dihydrochloride (AAPH), acetic acid (anhydrous), acetylcholine iodide, acetylcholinesterase, bovine serum albumin (BSA), caffeic acid, catechin, cinnamic acid, dinitro salicylic acid, epicatechin, ethanol, ethidium bromide, ferric chloride (FeCl₃), ferulic acid, gallic acid, hydrogen peroxide (H₂O₂), L-tyrosine, mushroom tyrosinase, N-(1-naphthyl)ethylenediamine dihydrochloride, *p*-coumaric acid, polysaccharide agarose, porcine α-amylase, rutin, sodium acetate buffer, sodium chloride, sodium dodecyl sulfate (SDS), sodium nitroprusside, sodium phosphate, sulfuric acid, syringic acid, Tris–HCl, and TPTZ (2,4,6-tri(2-pyridyl)-*s*-triazine).

### 2.3 Plant material extraction

Polyphenolic compounds were extracted from 18 varieties of date pits using a Dionex ASE 350 system (Thermo Fisher Scientific, USA). For each variety, 4 g of the sample was processed in 11 mL stainless steel extraction cells, with extracts collected in 40 mL amber vials. The extraction parameters were set as follows: pressure at 1500 psi, temperature at 25 °C, a 5-min static time, and 4 cycles of 4 min each. After each cycle, a 90-s nitrogen purge and a 75 % flush with fresh solvent were performed. The extraction solvent consisted of a methanol-water mixture (50:50, *v*/v), with the water component acidified to pH 2 using concentrated hydrochloric acid (HCl). This acidification enhanced the solubility of phenolic compounds, facilitating targeted extraction. The process was repeated for multiple batch experiments to ensure consistency and reproducibility (Habib, El-Gendi, et al., 2023).

### 2.4 HPLC analysis of phenolic acids and flavonoids

The quantification of phenolic acids and flavonoids in 18 distinct date fruit extract samples was conducted using a Waters HPLC system. The system comprised a 1525 Binary Pump, a 2487 Dual Absorbance UV Detector, and a 717 Plus Autosampler, all controlled by Breeze software (version 1.15) (Waters Corporation, Milford, MA, USA). Separation was achieved using a Waters XSelect HSS C18 column (5 μm, 4.6 mm × 150 mm). The mobile phases consisted of (A) 1 % acetic acid in water and (B) acetonitrile. A linear gradient elution program was applied, starting at 5 % B and gradually increasing to 95 % B over 80 min. The gradient was then rapidly reduced over 5 min, followed by an equilibration cycle for subsequent injections. The flow rate was maintained at 0.7 mL/min, with an injection volume of 20 μL. UV detection was performed at two wavelengths, 280 nm, and 330 nm, to measure absorbance. All analyses were carried out under ambient laboratory conditions (Alqahtani et al., 2024).

### 2.5 Antioxidant activity

#### 2.5.1 DPPH radical scavenging assay

The DPPH (2,2-diphenyl-1-picrylhydrazyl) radical scavenging activity of the 18 date pit extracts was evaluated using a modified version of a previously reported method (Alqahtani et al., 2024). In brief, 30 μL of each extract was mixed with 300 μL of a 90 μM DPPH methanolic solution and diluted with 570 μL of 80 % methanol. The mixture was incubated in the dark for 30 min, and absorbance was measured at 517 nm.

#### 2.5.2 ABTS radical scavenging assay

The ABTS radical scavenging activity was evaluated using a previously established method (Platat & Habib, 2014). A working ABTS solution was prepared by mixing equal volumes of 7 mM ABTS and 2.4 mM potassium persulfate stock solutions, followed by incubation in the dark at room temperature for 14 h. The solution was then diluted with methanol (1 mL ABTS solution to 60 mL methanol) to achieve an absorbance of 0.706 ± 0.02 at 734 nm. For the assay, 1 mL of each sample extract was mixed with 1 mL of the diluted ABTS solution and reacted for 7 min. Absorbance was measured at 734 nm, and the percentage inhibition, representing ABTS radical scavenging activity, was calculated using the formula:

(1) $$\%\text{Inhibition}=\left[\left(\mathrm{Abs}\;\text{control}–\mathrm{Abs}\;\text{sample}\right)/\mathrm{Abs}\;\text{control}\right]\times 100$$

where *Abs control* is the absorbance of the ABTS radical in methanol, and *Abs sample* is the absorbance of the ABTS radical solution mixed with the sample extract.

#### 2.5.3 Superoxide radical scavenging activity

The superoxide radical (O₂^−^) scavenging activity of the 18 date fruit extracts was assessed using a previously established method (Platat & Habib, 2014). Superoxide radicals were generated through the autoxidation of pyrogallol. In a 96-well microplate, 80 μL of each extract was combined with 80 μL of 50 mM Tris-HCl buffer (pH 8.3) containing 1 mM EDTA, followed by the addition of 40 μL of 1.5 mM pyrogallol in 10 mM HCl. The rate of pyrogallol polymerization (ΔA/min) was monitored by measuring the increase in absorbance at 420 nm over 4 min at room temperature. A blank was prepared by replacing the extract with a Tris-HCl buffer. All assays were conducted in triplicate, and the scavenging activity was calculated using Eq. (1), as given above.

#### 2.5.4 FRAP assay

The FRAP assay was conducted on 18 date fruit extracts using a previously established method (Habib et al., 2013). The FRAP reagent was prepared fresh by combining 10 mM TPTZ in 40 mM HCl, 20 mM FeCl₃, and 0.3 M acetate buffer (pH 3.6) in a 1:1:10 (*v*/v/v) ratio. For the assay, 1 mL of each sample was mixed with 2 mL of the FRAP reagent and incubated at 37 °C for 30 min. Absorbance was measured at 593 nm using a Varian CARY 50 Scan UV–VIS Spectrophotometer equipped with a Cary 50 Microplate Reader Accessory (Varian, Inc., Walnut Creek, CA, USA). Deionized water was used as the blank, and quantification was based on a FeSO₄ standard curve. Results were expressed as μmol Fe (II) equivalents.

### 2.6 Evaluation of enzyme inhibitory activities of date pits extracts

#### 2.6.1 Assessment of Tyrosinase inhibition by date pits extracts

Tyrosinase inhibition activity was measured spectrophotometrically (Habib et al., 2021). A mixture of 150 μL of sodium phosphate buffer (0.1 M, pH 6.8), 10 μL of date extract, and 20 μL of tyrosinase (50 units/mL) was incubated at 37 °C for 10 min. Then, 20 μL of L-tyrosine (1 mg/mL) was added, and the mixture was incubated for another 10 min. Absorbance was measured at 475 nm, and the inhibition percentage was calculated using Eq. (1), as given above.

#### 2.6.2 Assessment of porcine α-amylase inhibition by date pits extracts

With slight modifications, the α-amylase inhibitory activity was determined using a starch‑iodine assay (Habib et al., 2021). In a microplate well, 50 μL of α-amylase (1 mg/mL in 0.1 M sodium phosphate buffer) was mixed with 25 μL of date extract and incubated at 37 °C for 10 min. Then, 50 μL of 0.1 % *w*/*v* soluble starch was added, and the mixture was incubated for another 10 min. The reaction was stopped with 25 μL of 1 M HCl, followed by the addition of 100 μL of iodine reagent (5 mM I₂ and 5 mM KI). Absorbance was measured at 620 nm, and the inhibition percentage was calculated using Eq. (1), as given above.

#### 2.6.3 Assessment of acetylcholinesterase inhibition by date pits extracts

The acetylcholinesterase (AChE) inhibitory activity of the 18 date pits extracts was evaluated using a previously described method (Habib et al., 2017). A reaction mixture containing 325 μL of 0.05 M Tris-HCl buffer (pH 8), 100 μL of each extract, and 25 μL of AChE (0.28 U/mL) was incubated at room temperature for 15 min. Then, 475 μL of 3 mM DTNB solution and 75 μL of 15 mM acetylcholine iodide were added, and the mixture was incubated for another 30 min. Absorbance was measured at 405 nm over 5 min, and the inhibition percentage was calculated.

### 2.7 Evaluation of protective effects against BSA oxidative damage

The protective effects of date pits extract against BSA oxidation induced by AAPH were evaluated (Habib et al., 2017). BSA (0.5 mg/mL) was incubated with 20 mM AAPH, with or without date extract, at 37 °C for 30 min. A control without AAPH was included. The mixtures were analyzed using SDS-PAGE (10 %) under reducing conditions, and gel images were captured using a ChemiDoc MV system (Bio-Rad, USA). Band intensities were quantified using Image Lab 4.1 Software (Bio-Rad, USA) to assess protein damage.

### 2.8 Assessment of DNA protective effects against free radical damage

The protective effects of date pits extract against free radical-induced DNA damage were evaluated using a previously described method (Habib, Platat, Meudec, et al., 2014). A reaction mixture containing 4 μL of date extract, 6 μL of 30 % H₂O₂, 6 μL of PBS buffer, and 0.2 μg of pBR322 plasmid DNA (dissolved in 2 μL of 50 mM PBS, pH 7.4) was prepared. The mixtures were irradiated using a UV transilluminator (TFM-26, UVP, Upland, CA, USA) at 25 °C for 5 min (25 W cm^−2^ at 312 nm). After irradiation, the samples were electrophoresed on a 0.8 % agarose gel and stained with ethidium bromide. Gel images were analyzed using Image Lab 4.1 software (Bio-Rad, Hercules, CA, USA).

### 2.9 Explainable machine learning framework for predictive modeling and feature interpretation

A structured regression framework using XGBoost was developed to analyze the dataset, which consisted of a 54 × 18 matrix (54 samples (*n* = 3 × 18 date varieties = 54) × 18 attributes, with 9 features and 9 targets). Among the 18 attributes, 9 were designated as features and 9 as targets. The dataset was split into an 8:2 training-to-test ratio, with the training set used to build the XGBoost regression models and the test set for evaluating predictive performance. Model accuracy served as the primary evaluation metric. Hyperparameter optimization was performed using a grid search, and the model with the best performance was selected for detailed analysis. The models were implemented in Python 3.10 on the Google Colab cloud platform. To interpret the models, Tree SHAP from the SHAP library was employed, enabling the visualization of feature importance and the contributions of individual predictors (Yang et al., 2024).

### 2.10 Statistical evaluation and data analysis

All experiments were conducted in triplicate (*n* = 3). Data were analyzed using SPSS for Windows (version 26, SPSS Inc., Chicago, IL, USA). Differences between sample means were assessed using one-way analysis of variance (ANOVA), with statistical significance set at *p* < 0.05. Tukey's post hoc test was applied for multiple comparisons. Results are reported as mean ± standard deviation (SD). Pearson correlation analysis was used to examine relationships between variables where applicable.

## 3 Results and discussions

### 3.1 Comparative analysis of phenolic compound profiles in date pit extracts varieties

The phytochemical analysis revealed significant variations in the phenolic profiles of date pit extracts across 18 varieties, which are presented in Table 1. Catechin was the most abundant phenolic compound, with Maghool exhibiting the highest content (358.48 ± 2.60 mg/100 g), followed by Sokkery (352.03 ± 0.42 mg/100 g) and Shishi (324.81 ± 5.45 mg/100 g). Barhe showed the lowest catechin content (86.13 ± 1.62 mg/100 g). Bomaan had the highest epicatechin content (16.72 ± 0.62 mg/100 g), while Sultana had the lowest (4.96 ± 0.34 mg/100 g). Khalas exhibited the highest gallic acid content (34.55 ± 0.48 mg/100 g), and Shishi had the lowest (7.43 ± 0.03 mg/100 g). Fard showed the highest syringic acid content (23.74 ± 0.16 mg/100 g), while Lulu had the lowest (7.47 ± 0.22 mg/100 g). Shishi had the highest *p-*coumaric acid content (19.12 ± 0.13 mg/100 g), and Barhe had the lowest (5.56 ± 0.14 mg/100 g). Khalas exhibited the highest ferulic acid content (49.64 ± 0.11 mg/100 g), and Barhe had the lowest (2.72 ± 0.13 mg/100 g). Maghool had the highest cinnamic acid content (18.49 ± 0.23 mg/100 g), and Shishi had the lowest (10.28 ± 0.18 mg/100 g). Dabbas had the highest caffeic acid content (16.62 ± 0.36 mg/100 g), and Maghool had the lowest (5.38 ± 0.17 mg/100 g). Shishi had the highest rutin content (13.53 ± 0.16 mg/100 g), and Raziz had the lowest (5.73 ± 0.07 mg/100 g). The findings of this study align with previous research on the polyphenolic content of date fruits, although some variations were observed (Osaili et al., 2024; Shi et al., 2024; Swaidan et al., 2023). The variation in phenolic profiles among the date pits varieties is likely influenced by a combination of genetic, environmental, and cultivation factors. Genetic diversity among cultivars can lead to inherent differences in the types and amounts of phenolic profiles. Environmental conditions, such as sunlight exposure, temperature, and water availability, can affect the biosynthesis and accumulation of phenolic profiles in date pits. Cultivation practices, including fertilization and irrigation regimes, can also influence the phenolic profiles of date pits. Furthermore, postharvest handling and storage conditions may play a role in preserving or degrading phenolic profiles present in the pits (Alam et al., 2023; Hosseiny et al., 2023; Ouamnina et al., 2024; Toubali & Meddich, 2024).

**Table 1:** Comparative Analysis of Phytochemical Profiles in Extracts of Diverse Date Pit Varieties.

	Gallic Acid	Syringic acid	*P*-Coumaric acid	Ferulic acid	Cinnamic acid	Caffeic acid	Rutin	Catechin	Epicatechin
Khalas	34.55 ± 0.48^**n**^	24.38 ± 0.19^**l**^	16.53 ± 0.29^**i**^	49.64 ± 0.11^**k**^	19.00 ± 0.12^**i**^	27.45 ± 0.32^**k**^	9.20 ± 0.14^**g**^	130.21 ± 0.64^**c**^	13.25 ± 0.54^**g**^
Barhe	16.38 ± 0.36^**f**^	16.34 ± 0.22^**g**^	5.56 ± 0.14^**a**^	2.72 ± 0.13^**a**^	15.27 ± 0.13^**f**^	12.64 ± 0.26^**i**^	6.22 ± 0.14^**ab**^	86.13 ± 1.62^**a**^	5.21 ± 0.22^**ab**^
Lulu	9.64 ± 0.45^**b**^	7.47 ± 0.22^**a**^	14.08 ± 0.15^**h**^	35.67 ± 5.75^**j**^	22.03 ± 0.25^**j**^	10.71 ± 0.23^**h**^	7.67 ± 0.27^**de**^	158.45 ± 2.25^**d**^	11.01 ± 0.08^**f**^
Shikat alkahlas	18.26 ± 0.19^**h**^	11.04 ± 0.18^**d**^	10.59 ± 0.34^**f**^	27.75 ± 0.26^**i**^	17.29 ± 0.24^**h**^	6.69 ± 0.23^**bc**^	6.33 ± 0.30^**ab**^	106.45 ± 2.42^**b**^	6.47 ± 0.10^**c**^
Sokkery	21.21 ± 0.20^**jk**^	17.58 ± 0.21^**h**^	9.60 ± 0.22^**e**^	37.52 ± 0.19^**j**^	16.54 ± 0.38^**g**^	6.30 ± 0.17^**b**^	6.37 ± 0.11^**b**^	352.03 ± 0.42^**l**^	9.51 ± 0.20^**e**^
Bomaan	28.72 ± 0.29^**m**^	15.56 ± 0.29^**f**^	16.35 ± 0.13^**i**^	15.72 ± 0.34^**de**^	13.72 ± 0.15^**d**^	7.38 ± 0.18^**cde**^	8.41 ± 0.47^**f**^	231.15 ± 1.15^**h**^	16.72 ± 0.62^**h**^
Sagay	20.40 ± 0.32^**ij**^	20.41 ± 0.33^**k**^	18.04 ± 0.17^**j**^	23.53 ± 0.34^**ghi**^	15.52 ± 0.32^**f**^	9.28 ± 0.17^**g**^	7.77 ± 0.14^**def**^	213.04 ± 0.95^**g**^	11.12 ± 0.18^**f**^
Shishi	7.43 ± 0.03^**a**^	8.52 ± 0.20^**b**^	19.12 ± 0.13^**k**^	25.72 ± 0.29^**hi**^	10.28 ± 0.18^**a**^	6.82 ± 0.05^**bcd**^	13.53 ± 0.16^**j**^	324.81 ± 5.45^**k**^	7.75 ± 0.10^**d**^
Maghool	17.33 ± 0.23^**g**^	9.36 ± 0.20^**c**^	9.47 ± 0.21^**e**^	10.57 ± 0.26^**bc**^	18.49 ± 0.23^**i**^	5.38 ± 0.17^**a**^	10.42 ± 0.25^**h**^	358.48 ± 2.60^**l**^	9.32 ± 0.44^**e**^
Sultana	22.63 ± 0.10^**l**^	20.52 ± 0.33^**k**^	7.48 ± 0.15^**c**^	19.57 ± 0.42^**efg**^	11.59 ± 0.08^**b**^	11.31 ± 0.27^**h**^	8.18 ± 0.19^**ef**^	242.55 ± 2.22^**i**^	4.96 ± 0.34^**a**^
Fard	16.46 ± 0.36^**f**^	23.74 ± 0.16^**l**^	6.34 ± 0.12^**b**^	13.48 ± 0.38^**cd**^	15.78 ± 0.03^**f**^	11.41 ± 0.20^**h**^	6.72 ± 0.15^**bc**^	169.76 ± 1.99^**e**^	6.07 ± 0.27^**bc**^
Maktoomi	21.46 ± 0.11^**k**^	16.30 ± 0.24^**g**^	7.80 ± 0.13^**c**^	7.48 ± 0.16^**b**^	12.68 ± 0.28^**c**^	8.52 ± 0.22^**f**^	8.37 ± 0.16^**f**^	175.83 ± 0.58 e	6.18 ± 0.21^**bc**^
Naptit saif	15.41 ± 0.37^**e**^	15.55 ± 0.28^**f**^	6.60 ± 0.16^**b**^	9.49 ± 0.28^**bc**^	10.63 ± 0.14^**a**^	6.84 ± 0.06^**bcd**^	7.31 ± 0.10^**cd**^	314.48 ± 5.21^**j**^	10.29 ± 0.56^**ef**^
Jabri	18.57 ± 0.20^**h**^	20.61 ± 0.26^**k**^	8.48 ± 0.18^**d**^	17.33 ± 0.28^**def**^	14.52 ± 0.23^**e**^	9.63 ± 0.32^**g**^	10.48 ± 0.17^**h**^	310.96 ± 1.27^**j**^	6.88 ± 0.08^**cd**^
Khodary	20.01 ± 0.18^**i**^	20.06 ± 0.15^**jk**^	8.43 ± 0.20^**d**^	16.85 ± 0.25^**de**^	11.45 ± 0.12^**b**^	7.59 ± 0.32^**e**^	8.22 ± 0.22^**ef**^	207.47 ± 0.73^**fg**^	12.84 ± 0.43^**g**^
Dabbas	17.85 ± 0.24^**gh**^	18.41 ± 0.16^**j**^	11.14 ± 0.23^**fg**^	22.26 ± 0.18^**gh**^	13.71 ± 0.16^**d**^	16.62 ± 0.3^**6j**^	11.54 ± 0.11^**i**^	106.32 ± 2.09^**b**^	6.28 ± 0.32^**c**^
Raziz	13.53 ± 0.22^**d**^	19.55 ± 0.30^**j**^	13.53 ± 0.27^**h**^	21.24 ± 0.23^**fg**^	11.52 ± 0.32^**b**^	7.46 ± 0.21^**de**^	5.73 ± 0.07^**a**^	201.57 ± 2.39^**f**^	4.45 ± 0.07^**a**^
Shabebe	11.63 ± 0.31^**c**^	13.51 ± 0.28^**e**^	11.47 ± 0.27^**g**^	23.56 ± 0.28^**ghi**^	10.55 ± 0.21^**a**^	6.79 ± 0.17^**bcd**^	7.67 ± 0.19^**de**^	159.48 ± 0.91^**d**^	7.55 ± 018^**d**^

Values are presented as mean ± standard deviation (mg/100 g of dates pits). Means that are labeled with different letters within each column are significantly different (*p* < 0.05).

### 3.2 Antioxidant activity

Extracts from 18 date pit varieties were assessed for antioxidant activity using multiple assays to provide a comprehensive evaluation of their antioxidant potential, given the complexity of the underlying mechanisms (Habib, El-Fakharany, Kheadr, & Ibrahim, 2022). This activity is largely attributed to the presence of phenolic compounds, which act as potent antioxidants. However, variations in antioxidant activity were observed, likely due to a combination of factors, including maturity, fertilizer use, season, geographic origin, growing conditions, soil type, storage conditions, diseases, and extraction methods (Habib, El-Fakharany, Souka, et al., 2022). These factors can influence the biosynthesis and accumulation of phenolic compounds, leading to variations in the phenolic profiles of date pits. Furthermore, genetic diversity among cultivars can contribute to inherent differences in the types and amounts of phenolic compounds present. Environmental conditions, such as sunlight exposure, temperature, and water availability, can also affect the biosynthesis and accumulation of phenolic compounds in date pits. Cultivation practices, including fertilization and irrigation regimes, can further influence the phenolic profiles of date pits. Additionally, postharvest handling and storage conditions may play a role in preserving or degrading phenolic compounds present in the pits (Alam et al., 2023; Hosseiny et al., 2023; Ouamnina et al., 2024; Toubali & Meddich, 2024). The discrepancies observed between our results and other findings in the literature are likely attributed to the complex interplay of these factors.

#### 3.2.1 DPPH radical scavenging assay

**Fig. 1:**
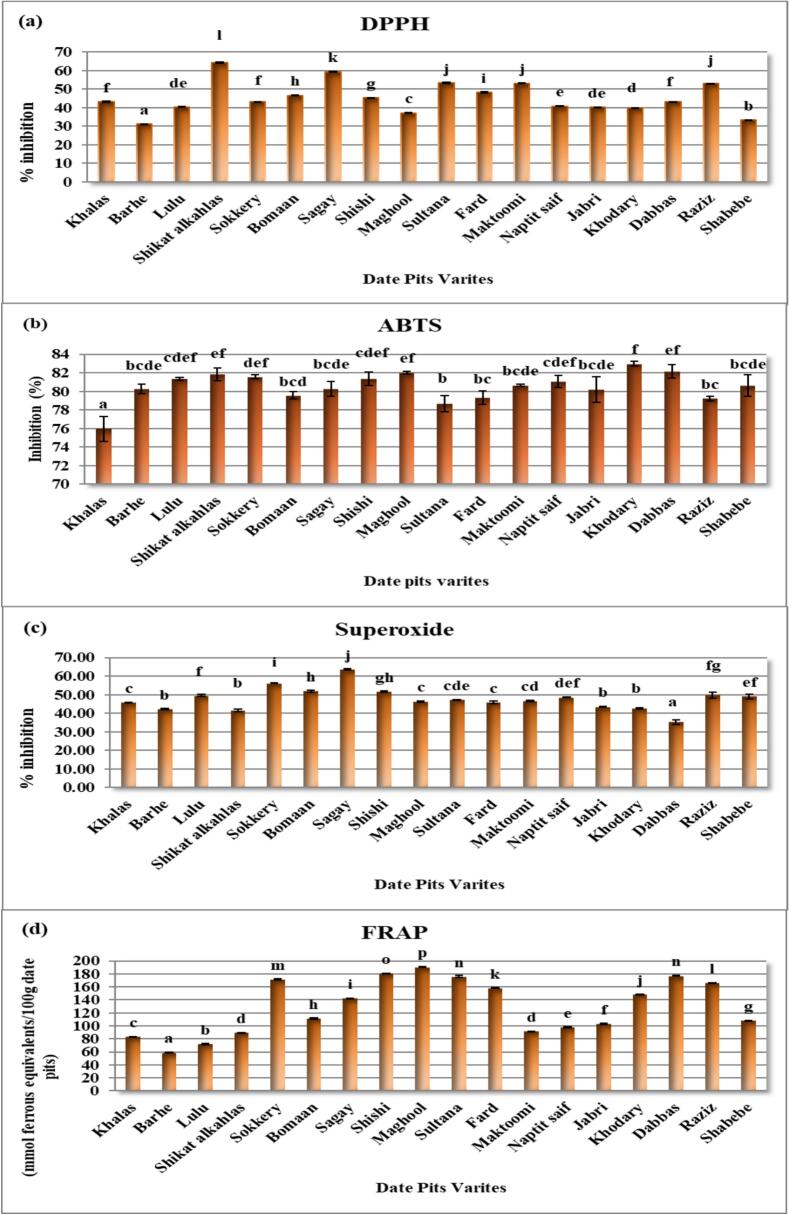
**(a-c),** Comparative Analysis of Antioxidant Activity in Date Pits Varieties Using DPPH, ABTS, Superoxide Scavenging, and FRAP Assays. (a) DPPH, (b) ABTS, (c) superoxide scavenging, and (d) FRAP assays. Bars represent mean ± SD. Different letters indicate significant differences (p < 0.05*).*

The antioxidant activity of date pits extracts from 18 varieties was evaluated using the DPPH radical scavenging assay. The results, expressed as a percentage of DPPH radical inhibition, are presented in Fig. 1a. Significant variations in antioxidant activity were observed among the different date pit varieties. The highest DPPH radical scavenging activity was exhibited by Shikat alkahlas (64.10 ± 0.32 %), followed by Sagay (59.26 ± 0.15 %) and Sultana (53.36 ± 0.15 %). Raziz and Maktoomi varieties also showed substantial antioxidant activity, recording 53.08 ± 0.05 % and 53.06 ± 0.11 % inhibition, respectively. In contrast, Barhe (31.07 ± 0.13 %) and Shabebe (33.20 ± 0.22 %) varieties displayed the lowest antioxidant activity. Statistical analysis revealed significant differences (*p* < 0.05) among the varieties.

The observed variations in DPPH radical scavenging activity among the date pits varieties suggest differences in their phytochemical composition, particularly in the content and type of antioxidant compounds. The high antioxidant activity of the Shikat alkahlas and Sagay varieties may be attributed to a higher concentration of phenolic compounds, flavonoids, or other radical scavenging molecules. These findings are consistent with previous studies that have reported significant antioxidant potential in date pits, correlating with their rich phytochemical profile (Osaili et al., 2024; Shi et al., 2024; Swaidan et al., 2023).

#### 3.2.2 Comparative Analysis of ABTS Radical Scavenging Activity in Diverse Date Pits Varieties

The antioxidant activity of date pits extracts from 18 varieties was assessed using the ABTS radical scavenging assay. The results, expressed as a percentage of ABTS radical inhibition, are presented in Fig. 1(b). Significant variations in antioxidant activity were observed among the different date pits varieties. The highest ABTS radical scavenging activity was exhibited by Khodary (82.98 ± 0.23 %), followed by Dabbas (82.16 ± 0.71 %) and Maghool (82.02 ± 0.15 %). Shikat alkahlas, Sokkery, Shishi, and Naptit saif varieties also showed substantial antioxidant activity, all registering above 81 % inhibition. In contrast, Khalas (75.94 ± 1.35 %) exhibited the lowest antioxidant activity.

The observed variations in ABTS radical scavenging activity among the date pits varieties suggest differences in their phytochemical composition, particularly in the content and type of antioxidant compounds capable of scavenging ABTS radicals. The high antioxidant activity of Khodary, Dabbas, and Maghool varieties may be attributed to a higher concentration of phenolic compounds, flavonoids, or other electron-donating molecules. These findings are consistent with previous studies that have reported significant antioxidant potential in date pits, correlating with their rich phytochemical profile (Habib, El-Fakharany, Kheadr, & Ibrahim, 2022; Osaili et al., 2024; Shi et al., 2024; Swaidan et al., 2023).

#### 3.2.3 Superoxide radical scavenging activity of date pits varieties

The superoxide radical scavenging activity of date pits extracts from 18 varieties was evaluated. The results, expressed as a percentage of superoxide radical inhibition, are presented in Fig. 1(c). Significant variations in superoxide radical scavenging activity were observed among the different date pit varieties. The highest superoxide radical scavenging activity was exhibited by Sagay (63.54 ± 0.35 %), followed by Sokkery (56.18 ± 0.27 %) and Bomaan (51.89 ± 0.51 %). Shishi also showed substantial activity (51.74 ± 0.33 %). In contrast, Dabbas (35.27 ± 1.28 %) exhibited the lowest superoxide radical scavenging activity.

The observed variations in superoxide radical scavenging activity among the date pits varieties suggest differences in their phytochemical composition, particularly in the content and type of antioxidant compounds capable of neutralizing superoxide radicals. The high activity observed in Sagay, Sokkery, and Bomaan varieties may be attributed to a higher concentration of phenolic compounds, flavonoids, or other molecules capable of donating electrons or hydrogen atoms to neutralize superoxide radicals. These findings are consistent with previous studies that have reported significant antioxidant potential in date pits, correlating with their rich phytochemical profile (Platat & Habib, 2014).

#### 3.2.4 Ferric reducing antioxidant power (FRAP) of date pits varieties

The ferric-reducing antioxidant power (FRAP) of date pits extracts from 18 varieties was evaluated. The results, expressed as mmol ferrous equivalents/100 g date pits, are presented in Fig. 1 (d). Significant variations in FRAP values were observed among the different date pits varieties. The highest FRAP value was exhibited by Maghool (190.57 ± 0.68 mmol ferrous equivalents/100 g), followed by Shishi (180.64 ± 0.38 mmol ferrous equivalents/100 g) and Dabbas (176.97 ± 0.78 mmol ferrous equivalents/100 g). Sultana and Sokkery varieties also showed substantial reducing power, registering 175.76 ± 1.46 and 171.20 ± 1.04 mmol ferrous equivalents/100 g, respectively. In contrast, Barhe (58.85 ± 0.23 mmol ferrous equivalents/100 g) exhibited the lowest FRAP value.

The observed variations in FRAP values among the date pits varieties suggest differences in their phytochemical composition, particularly in the content and type of antioxidant compounds capable of reducing ferric ions to ferrous ions. The high FRAP values observed in Maghool, Shishi, and Dabbas varieties may be attributed to a higher concentration of phenolic compounds, flavonoids, or other electron-donating molecules. These findings are consistent with previous studies that have reported significant antioxidant potential in date pits, correlating with their rich phytochemical profile (Osaili et al., 2024; Shi et al., 2024; Swaidan et al., 2023)

### 3.3 Enzyme inhibitory activities of date pits extracts

#### 3.3.1 Tyrosinase inhibitory activity of date pits extracts varieties

**Fig. 2:**
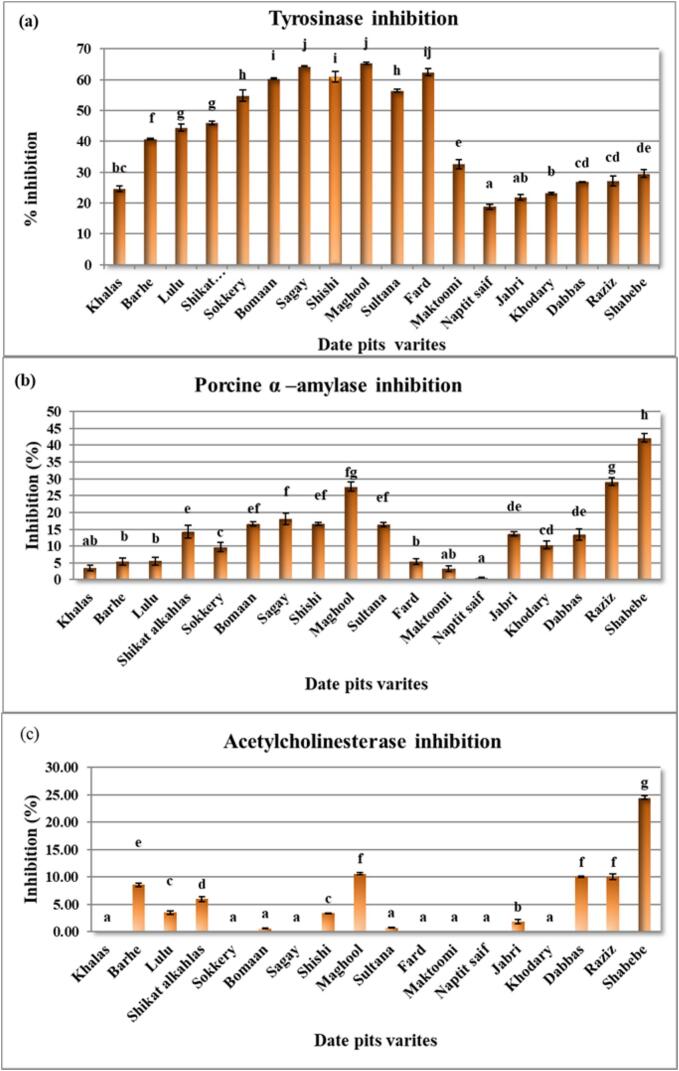
**(a-c).** Inhibitory Activities of Date Pit Extracts from Various Varieties Against Tyrosinase, Porcine α-Amylase, and Acetylcholinesterase. Inhibitory activities of date pit extracts from different varieties against (a) tyrosinase, (b) porcine α-amylase, and (c) acetylcholinesterase, expressed as percentage inhibition. Data are presented as mean ± standard deviation. Letters above bars indicate significant differences (*p* < 0.05) between treatments.

The tyrosinase inhibitory activity of date pit extracts from 18 varieties was investigated, revealing significant variations (*p* < 0.05) across the varieties (Fig. 2 a). Maghool exhibited the highest inhibitory activity (65.40 ± 0.39 %), followed by Sagay (64.39 ± 0.13 %) and Fard (62.50 ± 1.10 %). Naptit saif showed the lowest activity (18.75 ± 0.73 %). These variations suggest differences in phytochemical composition, with higher inhibitory activity potentially attributed to a greater abundance of tyrosinase-inhibiting compounds, such as phenolics and flavonoids. The observed differences underscore the importance of selecting specific date pit varieties for applications related to tyrosinase inhibition. The inhibition of tyrosinase by date pit extracts could involve various mechanisms, including competitive inhibition, non-competitive inhibition, chelation of copper ions, or direct interaction with the catalytic site. Further research is needed to identify the specific bioactive compounds responsible for this activity, to elucidate their mechanisms of action, and to explore their potential applications in cosmetic formulations or food preservation.

#### 3.3.2 Α-Amylase inhibitory activity of date pits extracts varieties

The α-amylase inhibitory activity of date pit extracts from 18 varieties was investigated, revealing significant variations (*p* < 0.05) across the varieties presented in Fig. 2 (b). Shabebe exhibited the highest inhibitory activity (42.21 ± 1.35 %), followed by Raziz (29.16 ± 1.16 %) and Maghool (27.65 ± 1.37 %). Naptit saif showed the lowest activity (0.54 ± 0.09 %). These variations suggest differences in phytochemical composition, with higher inhibitory activity potentially attributed to a greater abundance of α-amylase-inhibiting compounds, such as phenolics and flavonoids. The observed differences underscore the importance of selecting specific date pit varieties for applications related to α-amylase inhibition. The inhibition of α-amylase by date pit extracts could involve various mechanisms, including competitive, non-competitive, mixed, or uncompetitive inhibition. Further research is needed to identify the specific bioactive compounds responsible for this activity, to elucidate their mechanisms of action, and to explore their potential therapeutic applications in managing conditions like type 2 diabetes.

#### 3.3.3 Acetylcholinesterase inhibitory activity of date pits extracts varieties

The acetylcholinesterase (AChE) inhibitory activity of date pit extracts from 18 varieties was investigated, revealing significant variations (*p* < 0.05) across the varieties (Fig. 2c). Shabebe exhibited the highest inhibitory activity (24.48 ± 0.34 %), followed by Maghool (10.59 ± 0.16 %), Raziz (10.02 ± 0.54 %), and Dabbas (9.98 ± 0.14 %), while Khalas, Sokkery, Sagay, Fard, Maktoomi, Naptit saif, and Khodary showed no detectable activity. These disparities likely reflect differences in phytochemical composition, with elevated inhibitory activity potentially linked to higher concentrations of AChE-interacting compounds, such as phenolics and alkaloids. The observed variability underscores the importance of variety selection for applications targeting AChE inhibition. Mechanistically, inhibition by date pit extracts may involve multiple pathways: (1) competitive inhibition, where bioactive compounds compete with acetylcholine for binding to the enzyme's active site; (2) non-competitive inhibition via binding to allosteric sites, inducing conformational changes that reduce catalytic efficiency; (3) mixed inhibition, combining competitive and non-competitive modes; or (4) direct interaction with residues of the catalytic triad (Ser-His-Glu), obstructing substrate access or hydrolysis. The predominant mechanism(s) may vary depending on the specific variety and its phytochemical profile, particularly the identity and concentration of bioactive constituents. Further research is warranted to isolate and characterize the compounds responsible for AChE inhibition, elucidate their precise molecular interactions (e.g., binding kinetics, structural modifications), and evaluate their therapeutic potential in managing cholinergic deficits associated with neurodegenerative disorders such as Alzheimer's disease.

### 3.4 Protective effects of date pits extracts from various varieties against BSA damage

**Fig. 3:**
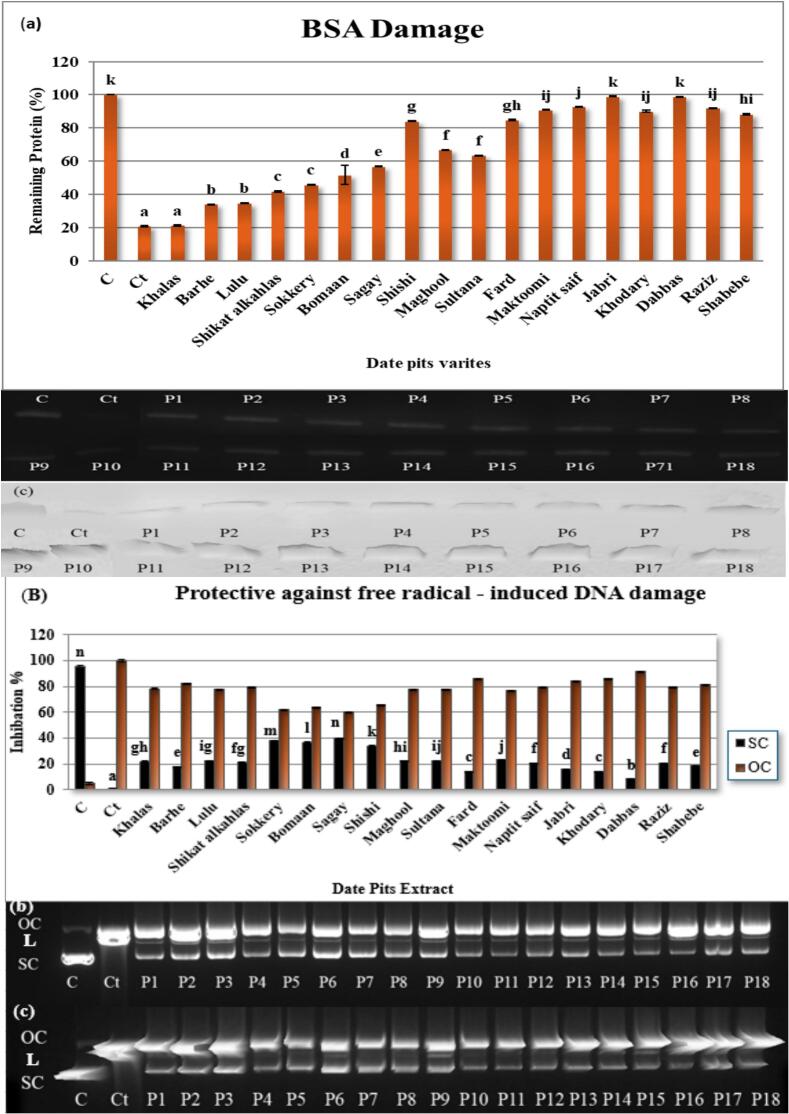
Protective effects of date pit extracts from various varieties against BSA damage and free radical-induced DNA damage. (A) Percentage of remaining protein in BSA after treatment with date pit extracts, showing protection against damage. (B) Percentage inhibition of free radical-induced DNA damage by date pit extracts, quantified as the reduction of open circular (OC) and linear (L) DNA forms, with the remaining supercoiled (SC) DNA shown as a percentage. (C) Representative agarose gel electrophoresis images of DNA showing the protective effects of date pit extracts. C = control (untreated), Ct = control treated with damaging agent, P1-P18 = date pit extracts from different varieties. Letters above bars in (A) and (B) indicate significant differences (*p* < 0.05) between treatments.

**Fig. 4:**
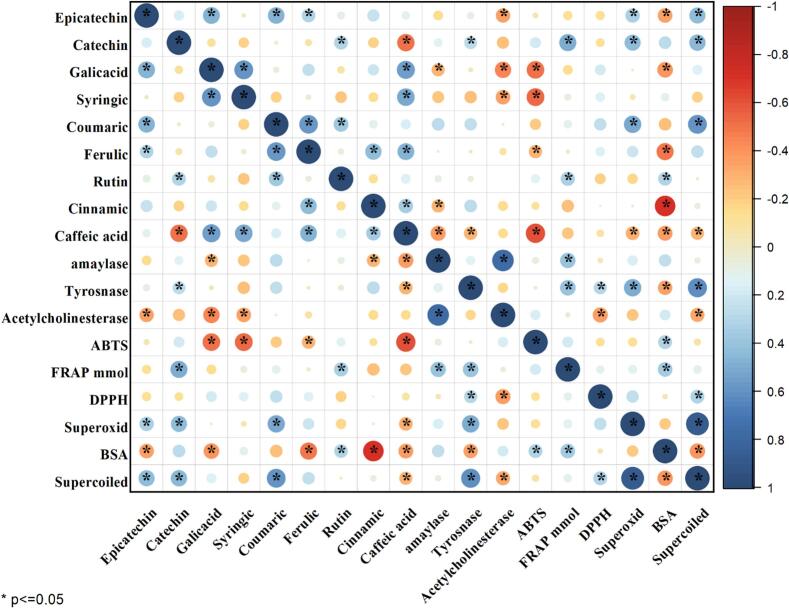
Heatmap Visualization of Correlations between Phytochemicals, Antioxidant Activities, Enzyme Inhibition, and Protective Effects against DNA and BSA Damage in Date Pit Extracts. The heatmap visually represents the correlation coefficients, with blue shades indicating positive correlations, red shades indicating negative correlations, and the size of the circles representing the strength of the correlation. The color scale helps quickly identify the magnitude and direction of relationships. Additionally, statistically significant values are denoted with an asterisk (*).

The protective effects of date pit extracts from 18 varieties against (BSA) damage were evaluated by measuring the percentage of remaining protein, as shown in Fig. 3(a). The untreated control (C) showed 100 % remaining protein, indicating no damage. The treated control (Ct) with the damaging agent exhibited a significant decrease to 20.78 ± 0.55 % remaining protein, confirming substantial BSA damage. Among the date pit extracts, Jabri and Dabbas showed the highest protective effects, with 98.95 ± 0.20 % and 98.83 ± 0.21 % remaining protein, respectively, indicating near-complete protection. Naptit saif, Raziz, and Maktoomi also demonstrated significant protection, with 92.78 ± 0.23 %, 92.08 ± 0.19 %, and 90.85 ± 0.18 % remaining protein, respectively. Khodary showed 90.16 ± 0.56 % and Shabebe had 88.34 ± 0.49 % remaining protein. Fard and Shishi exhibited moderate protection with 84.90 ± 0.37 % and 84.18 ± 0.28 % remaining protein. Maghool and Sultana showed moderate protection with 66.80 ± 0.25 % and 63.30 ± 0.34 % remaining protein. Sagay exhibited 56.84 ± 0.24 % remaining protein. Bomaan had 51.61 ± 5.75 % remaining protein. Sokkery and Shikat alkahlas showed 45.74 ± 0.24 % and 41.85 ± 0.38 % remaining protein. Lulu and Barhe exhibited 34.70 ± 0.37 % and 33.98 ± 0.24 % remaining protein. Khalas showed the lowest protection with 21.19 ± 0.35 % remaining protein. Khalas's low protection may correlate with its lower catechin (130.21 mg/100 g) and epicatechin (13.25 mg/100 g) content compared to high-performing varieties. (See Fig. 4.)

The damaging agent significantly reduced the remaining protein, confirming BSA damage. Date pit extracts exhibited varying degrees of protection against this damage, with Jabri and Dabbas showing near-complete protection, while others like Khalas offered minimal protection. This variation suggests differences in the concentration and type of protective compounds present in different date pit varieties. Further research is needed to identify these compounds and explore their potential therapeutic applications.

### 3.5 Protective effects of date pits extracts from various varieties against DNA damage

Fig. 3(B) represents the protective effects of date pit extracts from 18 varieties, along with controls, which were assessed against free radical-induced DNA damage by quantifying the percentage of remaining supercoiled DNA (SC). The untreated control (C) exhibited 95.36 ± 0.64 % supercoiled DNA, while the treated control (Ct) showed 4.64 ± 0.64 %. Among the date pit extracts, Sagay demonstrated the highest protective effect with 39.99 ± 0.12 % supercoiled DNA remaining, followed closely by Sokkery (38.01 ± 0.13 %) and Bomaan (36.57 ± 0.19 %). Shishi also showed notable protection with 33.92 ± 0.07 % supercoiled DNA. Conversely, Dabbas exhibited the lowest protective effect with only 8.13 ± 0.10 % supercoiled DNA remaining. Other varieties, including Khalas, Lulu, Maghool, Sultana, Maktoomi, and Raziz, showed moderate protective effects, ranging from 20.41 ± 0.09 % to 22.95 ± 0.07 % supercoiled DNA.

This study provides the first evidence of the protective effects of date pit extracts against free radical-induced DNA damage, demonstrating varying degrees of protection across different varieties. The observed shift from supercoiled to open circular DNA form (Habib, El-Fakharany, et al., 2023) confirms damage by free radicals, likely due to single-strand breaks caused by nucleases or oxidative damage (Cao et al., 2024; Xin et al., 2024). The extracts' ability to preserve supercoiled DNA suggests the presence of protective compounds, consistent with the known antioxidant properties of date pits (Habib, Al Meqbali, Kamal, et al., 2014; Habib et al., 2017; Habib, El-Fakharany, Souka, et al., 2022). The variation in effectiveness across extracts likely reflects differences in the type and amount of bioactive compounds, such as polyphenols and flavonoids (El Monfalouti & Eddine Kartah, 2024; Habib, El-Fakharany, Kheadr, & Ibrahim, 2022; Habib, El-Fakharany, Souka, et al., 2022; Vicidomini et al., 2024). While the precise mechanism remains unclear, this study highlights the potential of date pit extracts as a source of DNA-protective agents. Further research is needed to identify the specific compounds and their mechanisms of action and to evaluate the in vivo effects. This study lays the foundation for future investigations into the therapeutic potential of date pit extracts in preventing DNA damage-related diseases.

### 3.6 Correlation analysis of phytochemicals and bioactivities

The Pearson correlation coefficients among various bioactive compounds (gallic acid, syringic acid, *p*-coumaric acid, ferulic acid, cinnamic acid, caffeic acid, rutin, catechin, and epicatechin) and their antioxidant and enzyme inhibitory activities (amylase, tyrosinase, acetylcholinesterase inhibition, ABTS, FRAP, DPPH, superoxide scavenging, BSA protection, and supercoiled DNA protection) are presented in Figure. Significant correlations (*p* < 0.05 or *p* < 0.01) were observed between several pairs of variables.

Gallic acid showed significant positive correlations with syringic acid (*r* = 0.587, *p* < 0.01), caffeic acid (*r* = 0.542, *p* < 0.01), and epicatechin (*r* = 0.474, *p* < 0.01), indicating potential co-occurrence or synergistic effects of these compounds. Conversely, gallic acid exhibited significant negative correlations with amylase inhibition (*r* = −0.288, *p* < 0.05), acetylcholinesterase inhibition (*r* = −0.443, *p* < 0.01), ABTS (*r* = −0.517, p < 0.01), and BSA damage protection (*r* = −0.399, *p* < 0.01). Meanwhile, syringic acid demonstrated a significant positive correlation with caffeic acid (*r* = 0.511, *p* < 0.01) and significant negative correlations with acetylcholinesterase inhibition (*r* = −0.348, *p* < 0.01) and ABTS (*r* = −0.529, *p* < 0.01). *p*-Coumaric acid showed significant positive correlations with ferulic acid (*r* = 0.573, *p* < 0.01), rutin (*r* = 0.369, *p* < 0.01), epicatechin (*r* = 0.461, *p* < 0.01), superoxide scavenging (*r* = 0.502, *p* < 0.01), and supercoiled DNA protection (*r* = 0.581, *p* < 0.01).

Furthermore, Ferulic acid was positively correlated with cinnamic acid (*r* = 0.437, *p* < 0.01) and caffeic acid (*r* = 0.467, *p* < 0.01) and negatively correlated with BSA damage protection (*r* = −0.481, *p* < 0.01). Caffeic acid had a significant negative correlation with catechin (*r* = −0.503, *p* < 0.01) and ABTS (*r* = −0.612, *p* < 0.01). Nevertheless, catechin showed significant positive correlations with rutin (*r* = 0.314, *p* < 0.05), FRAP (*r* = 0.482, *p* < 0.01), superoxide scavenging (*r* = 0.432, *p* < 0.01), and supercoiled DNA protection (*r* = 0.443, *p* < 0.01). Amylase inhibition was positively correlated with acetylcholinesterase inhibition (*r* = 0.770, *p* < 0.01) and FRAP (*r* = 0.388, *p* < 0.01). Tyrosinase inhibition showed significant positive correlations with FRAP (*r* = 0.397, *p* < 0.01), DPPH (*r* = 0.287, *p* < 0.05), superoxide scavenging (*r* = 0.493, *p* < 0.01), and supercoiled DNA protection (*r* = 0.605, *p* < 0.01).

The correlation analysis revealed complex relationships between the bioactive compounds and the observed antioxidant and enzyme inhibitory activities in date pit extracts. The significant positive correlations among certain phenolic compounds suggest that they may be acting synergistically to contribute to the overall bioactivity. Additionally, the negative correlations observed between gallic acid and several antioxidant assays (ABTS, BSA) and enzyme inhibition (amylase, Acetylcholinesterase) indicate that while gallic acid may contribute to some activities, other compounds or mechanisms might be more influential in these specific assays. Similarly, the negative correlations between caffeic acid and catechin suggest potential competitive interactions or different mechanisms of action. The positive correlations of *p*-coumaric acid, catechin, and tyrosinase with various antioxidant and DNA protection assays highlight their significant contributions to these activities. The strong positive correlation between amylase and acetylcholinesterase inhibition suggests a potential link between these activities, possibly through shared mechanisms or compounds. The negative correlation of ferulic acid and cinnamic acid with BSA damage protection and the strong negative correlation of cinnamic acid with BSA protection indicates that these compounds might not be the primary contributors to BSA protection in these extracts.

The high correlation coefficients observed between superoxide scavenging and supercoiled DNA protection, as well as tyrosinase inhibition and supercoiled DNA protection, suggest that these activities are closely related and may be mediated by similar compounds or mechanisms.

Overall, the correlation analysis provides valuable insights into the relationships between bioactive compounds and their biological activities in date pit extracts. These findings can guide further studies aimed at identifying specific compounds responsible for the observed activities and understanding their mechanisms of action.

### 3.7 Identifying key features of date pit extracts for bioactivity prediction using XGBoost

#### 3.7.1 XGBoost for date pit multi-target bioactivity

**Fig. 5:**
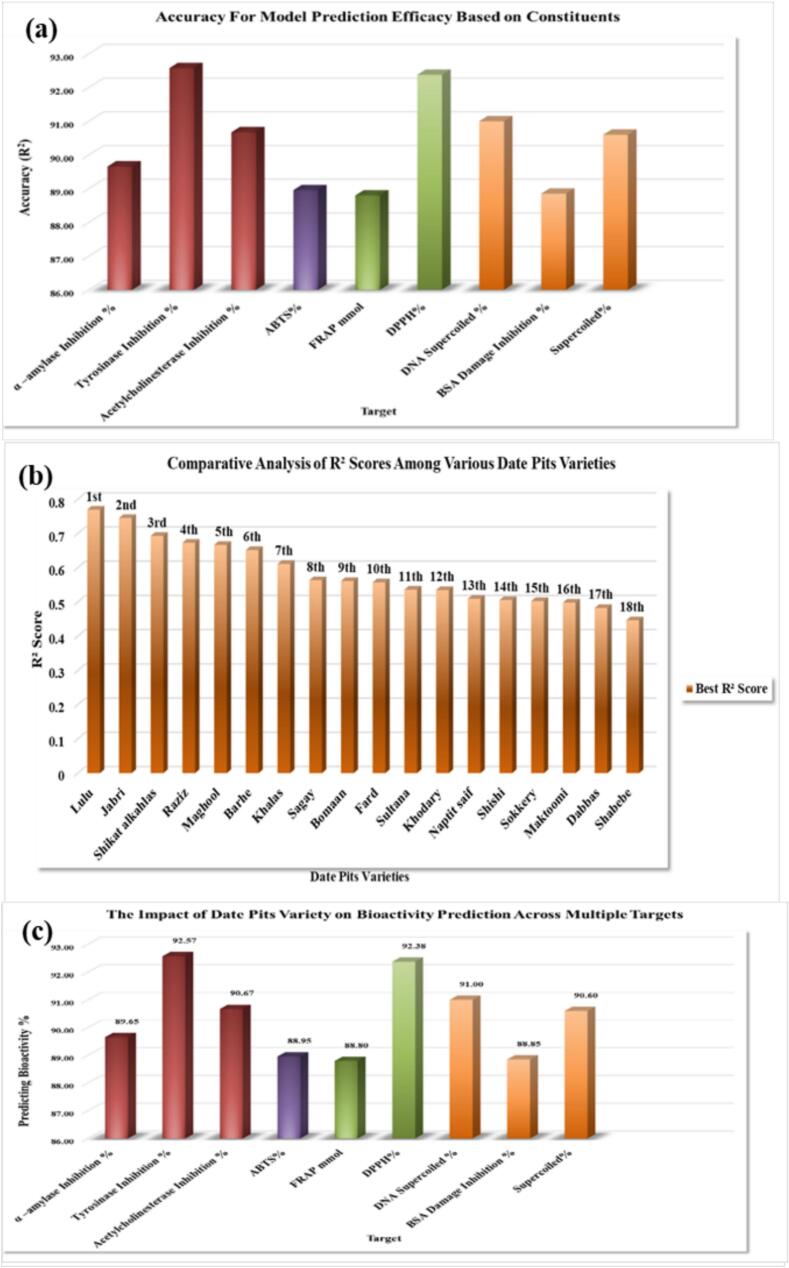
Performance of XGBoost Model in Predicting Bioactivity of Date Pit Extracts: (a) Accuracy for Different Bioactivity Targets, (b) Comparative R^2^ Scores Across Date Pit Varieties, and (c) Impact of Date Pit Variety on Bioactivity Prediction Accuracy.

The predictive accuracy of the XGBoost machine learning model was evaluated across nine distinct bioactivity targets using features derived from date pit extracts (Yang et al., 2024). As demonstrated in Fig. 5a, the model demonstrated high accuracy in predicting various outcomes, including enzyme inhibition (α-amylase, tyrosinase, acetylcholinesterase), antioxidant activity (ABTS, FRAP, DPPH), DNA supercoiling inhibition, and BSA damage inhibition. The accuracy ranged from 88.80 % to 92.57 % across all targets. Specifically, the highest accuracy was achieved in predicting tyrosinase inhibition (92.57 %), followed closely by ABTS radical scavenging activity (92.38 %) and DNA supercoiling inhibition (91.00 %). The lowest accuracy was observed in predicting DPPH radical scavenging activity (88.80 %) and BSA damage inhibition (88.85 %).

The consistently high accuracy of the XGBoost model across diverse bioactivity targets suggests that the features derived from date pit extracts are robust predictors of these outcomes (Joshi et al., 2024; Kim et al., 2024). This indicates that the phytochemical composition of date pits, as captured by the features used in the model, is strongly associated with a wide range of biological activities. The high accuracy observed for enzyme inhibition predictions (α-amylase, tyrosinase, acetylcholinesterase) suggests that the model effectively captures the relationships between specific date pit constituents and their ability to inhibit these enzymes (M'hamdi et al., 2024). This has significant implications for the potential use of date pit extracts in managing conditions related to these enzymes, such as diabetes and neurodegenerative diseases.

The strong performance in predicting antioxidant activities (ABTS, FRAP, DPPH) further validates the antioxidant potential of date pit extracts and demonstrates the model's ability to accurately predict these activities based on the input features. The high accuracy in predicting DNA supercoiling and BSA damage inhibition suggests that date pit extracts may possess protective effects against DNA damage and protein oxidation. These findings highlight the potential of date pit extracts as a source of bioactive compounds with diverse therapeutic applications (Jung et al., 2024; Li et al., 2024).

The slight variations in accuracy across different targets may be attributed to differences in the complexity of the underlying mechanisms or the specific features that are most relevant for each target (Singh, Varma, et al., 2024).

For example, predicting enzyme inhibition might rely on a different set of features compared to predicting antioxidant activity. Further analysis of feature importance could provide insights into the specific compounds or properties of date pit extracts that are most predictive for each target (Jung et al., 2024).

The high overall accuracy of the XGBoost model demonstrates its potential as a powerful tool for predicting the bioactivity of date pit extracts and for guiding the selection of specific extracts for further investigation or application. This machine-learning approach can significantly accelerate the discovery and development of novel bioactive compounds from date pits.

#### 3.7.2 XGBoost model performance for date pit bioactivity

The predictive performance of the XGBoost machine learning model in predicting bioactivity outcomes from date pit extracts was assessed for 18 different date pit varieties. The results, expressed as R^2^ scores, are presented in Fig. 5b. Significant variations in model performance were observed across the varieties. The highest R^2^ score was achieved for ‘Lulu’ (0.7695), indicating the best predictive performance for this variety. ‘Jabri’ (0.7452) and ‘Shikat alkahlas’ (0.6929) also showed relatively high R^2^ scores. The lowest R^2^ score was observed for ‘Shabebe’ (0.4463), suggesting the poorest predictive performance for this variety.

The XGBoost model's ability to predict bioactivity outcomes from date pit extracts varied across 18 different varieties, with R^2^ scores ranging from 0.4463 (Shabebe) to 0.7695 (Lulu). Higher R^2^ scores for varieties like Lulu, Jabri, and Shikat alkahlas suggest a strong relationship between phytochemical composition and bioactivity, potentially due to a higher concentration or unique combination of bioactive compounds effectively captured by the model's features. Conversely, lower scores for other varieties may reflect greater variability in phytochemical composition, the presence of unidentified compounds, complex interactions, or limitations in sample size or quality. These findings underscore the importance of considering variety-specific characteristics when developing predictive models for bioactivity, prompting further investigation into the phytochemical profiles and biological activities of individual varieties to optimize model performance and explore alternative feature sets or model architectures (Ortiz et al., 2024; Sydney Anuyah et al., 2024).

#### 3.7.3 The impact of date pits variety on bioactivity prediction across multiple targets

The impact of date pit variety on bioactivity prediction across multiple targets was assessed using a machine-learning model (Fig. 5c). The model's predictive accuracy, expressed as percentages, varied significantly across different bioactivity assays. The highest accuracy was observed for tyrosinase inhibition (85.46 %), indicating a strong influence of date pit variety on predicting this specific enzyme inhibition. Conversely, the lowest accuracy was seen in predicting superoxide inhibition (3.09 %), suggesting that date pit variety alone may not be a significant predictor of this particular antioxidant activity. Moderate to high accuracies were observed for other targets, including α-amylase inhibition (72.03 %), DPPH radical scavenging (67.54 %), acetylcholinesterase inhibition (54.94 %), DNA open circular inhibition (56.62 %), DNA supercoiled inhibition (56.58 %), anti-cancer activity (54.17 %), BSA damage inhibition (58.59 %), ABTS radical scavenging (56.61 %), and FRAP reducing power (7.04 %). These results suggest that date pit variety plays a substantial role in predicting certain bioactivities, particularly enzyme inhibition and radical scavenging, while its influence is less pronounced for others (Djaoudene et al., 2024). The observed variations in predictive accuracy highlight the complex relationship between date pit variety and bioactivity, potentially reflecting differences in phytochemical composition, compound interactions, or the specific mechanisms involved in each assay (Singh, Gupta, et al., 2024). Further investigation into the specific compounds responsible for these activities and their variety-dependent variations is warranted to fully understand the significance of date pit variety in bioactivity prediction.

#### 3.7.4 Feature importance analysis

**Fig. 6:**
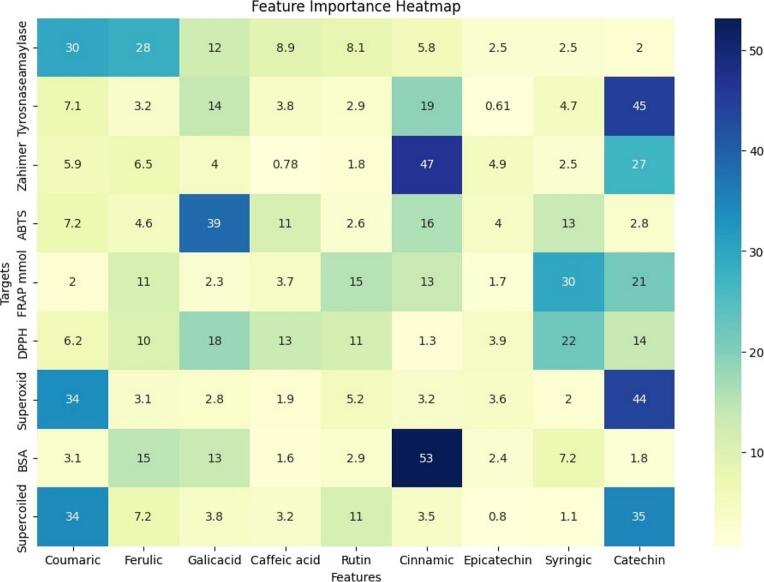
Heatmap of Feature Importance Analysis of Date Pit Extract Constituents for Predicting Bioactivity Across Multiple Targets Using XGBoost.

Fig. 6 illustrated a heatmap visualization that was generated to illustrate the relative importance of nine bioactive constituents from date pit extracts (coumaric acid, ferulic acid, gallic acid, caffeic acid, rutin, cinnamic acid, epicatechin, syringic acid, and catechin) in predicting bioactivity across ten targets using the XGBoost machine learning model. The heatmap displays feature importance scores, with darker shades indicating higher importance. Catechin exhibited the highest importance for predicting acetylcholinesterase, α-amylase inhibition, and superoxide inhibition. Cinnamic acid demonstrated the highest importance for predicting BSA damage inhibition. Rutin showed the highest importance for predicting FRAP-reducing power. Gallic acid was most influential in predicting superoxide inhibition, while epicatechin showed moderate importance across several targets, particularly tyrosinase inhibition. *P*-coumaric acid and caffeic acid exhibited relatively lower importance across most targets.

The feature importance heatmap reveals the differential contributions of specific date pit extract constituents to predicting bioactivity across various targets (Singh, Varma, et al., 2024). Catechin emerged as a key predictor for multiple bioactivities, particularly those related to neurodegenerative diseases and antioxidant mechanisms. This suggests that catechin, or compounds closely associated with it, plays a crucial role in conferring these biological activities. The high importance of cinnamic acid for predicting BSA damage inhibition indicates its potential role in protecting proteins from oxidative stress.

The significance of rutin in predicting FRAP activity aligns with its known antioxidant properties. The observed variations in feature importance across targets highlight the target-specific nature of bioactive compound contributions (Xing et al., 2023). For example, while catechin is crucial for predicting acetylcholinesterase, it is less influential in predicting DPPH radical scavenging. Similarly, gallic acid is highly important for predicting superoxide inhibition but has a lower impact on other targets. These findings suggest that different mechanisms of action are likely involved for each bioactivity, with specific compounds playing dominant roles (Xing et al., 2023). The relatively lower importance of coumaric acid and caffeic acid across most targets may indicate that these compounds, while present in the extracts, do not significantly contribute to the predicted bioactivities or that their effects are mediated through interactions with other compounds. The heatmap provides a valuable tool for identifying key bioactive constituents and guiding further research focused on isolating and characterizing these compounds. Understanding the target-specific contributions of these constituents can aid in developing targeted applications of date pit extracts for various health benefits. Further studies are needed to validate these findings and to explore the synergistic effects of these compounds in conferring bioactivity.

## 4 Conclusion

This study highlights the remarkable variability in bioactive compounds and functional properties among 18 date pit varieties, with Maghool, Sagay, and Shikat alkahlas emerging as particularly potent in antioxidant and enzyme-inhibitory activities. The integration of biochemical assays and explainable ML revealed catechin, rutin, and cinnamic acid as critical contributors to DNA protection, radical scavenging, and enzyme inhibition. The XGBoost model's high predictive accuracy (up to 92.57 %) underscores its utility in bioactivity prediction, enabling targeted extraction of bioactive-rich varieties. However, the precise mechanisms of action and in vivo efficacy remain uncharacterized. Future research should focus on isolating and validating specific bioactive compounds, furthermore, elucidating molecular pathways in preclinical models, nevertheless, translating findings into clinical or industrial applications, such as functional foods or therapies for oxidative stress-related disorders. This work establishes a foundation for leveraging date pits as a sustainable resource in precision nutrition and preventive medicine.

## Declaration of Generative AI and AI-assisted technologies in the writing process statement

During the preparation of this work the authors used ‘Quillbot’ to paraphrase and improve readability and language. After using this tool, the authors reviewed and edited the content as needed and took full responsibility for the content of the publication.

## CRediT authorship contribution statement

**Nashi K. Alqahtani:** Writing – review & editing, Supervision, Resources, Project administration, Funding acquisition. **Tareq M. Alnemr:** Writing – review & editing, Methodology, Formal analysis, Data curation. **Hoda A.S. Farag:** Writing – review & editing, Methodology, Formal analysis. **Rania Ismail:** Writing – review & editing, Visualization, Validation, Software, Methodology, Data curation. **Hosam M. Habib:** Writing – review & editing, Writing – original draft, Visualization, Validation, Project administration, Investigation, Conceptualization.

## Funding

This study was funded by the Deanship of Scientific Research, Vice Presidency for Graduate Studies and Scientific Research, King Faisal University, Saudi Arabia (Grant Number: KFU251477).

## Declaration of competing interest

The authors declare that they have no known competing financial interests or personal relationships that could have appeared to influence the work reported in this paper.

## Data Availability

No data was used for the research described in the article.
